# Biomarkers of treatment response in patients with progressive multiple sclerosis treated with high‐dose pharmaceutical‐grade biotin (MD1003)

**DOI:** 10.1002/brb3.1998

**Published:** 2020-12-13

**Authors:** Nicolas Collongues, Jens Kuhle, Charidimos Tsagkas, Julien Lamy, Nicolas Meyer, Christian Barro, Katrin Parmar, Michael Amann, Jens Wuerfel, Ludwig Kappos, Thibault Moreau, Jerome de Seze

**Affiliations:** ^1^ Department of Neurology University Hospital of Strasbourg Strasbourg France; ^2^ Neurological Clinic and Polyclinic Departments of Medicine, Clinical Research and Biomedical Engineering University Hospital Basel University of Basel Basel Switzerland; ^3^ Medical Image Analysis Centre Basel and Department of Biomedical Engineering University of Basel Basel Switzerland; ^4^ ICube Université de Strasbourg‐CNRS University of Strasbourg Strasbourg France; ^5^ GMRC, Service de Santé Publique University Hospital of Strasbourg Strasbourg France; ^6^ University of Basel Basel Switzerland; ^7^ Department of Neurology University Hospital of Dijon Dijon France

**Keywords:** brain volume, MD1003, multiple sclerosis, neurofilament light chain, progressive form, spinal cord volume

## Abstract

**Background:**

High‐dose pharmaceutical‐grade biotin (MD1003) has positive effects on disability in progressive multiple sclerosis (PMS), but its mechanism of action remains unclear. The objective of our study was to quantify the effect of MD1003 in patients with PMS, using clinical response, plasma neurofilament light chain (pNfL) levels, and brain (BV) or cervical spinal cord volume (CSCV).

**Materials and methods:**

Forty‐eight patients with PMS newly treated with MD1003 were followed during one year. Patients were assessed clinically using the Expanded Disability Status Scale (EDSS), the nine‐hole peg test (9HPT), and the 25‐foot walk time (25FWT). CSCV was quantified using CORDIAL software and BV using SIENA or SIENAX. We measured pNfL level using SIMOA at several time points. Bayesian linear and logistic regressions were used to evaluate potential prognostic factors.

**Results:**

Treatment response, defined as a significant decrease of EDSS, 25FWT, or 9HPT at 1 year, was observed in 13 patients (27%). A gain of volume was noted in 7/24 patients for brain and in 10/19 patients for cervical spinal cord. The strongest predictors of poor treatment response were a high pNfL level at MD1003 onset (OR 0.96; 95% CI [0.91; 1]), high age at MS onset (OR 0.95; 95% CI [0.89; 1.01]), and an increase in brain lesion load during MD1003 treatment (OR 0.81; 95% CI [0.55; 1.05]).

**Conclusions:**

MD1003 treatment was associated with clinical, BV, and CSCV improvement at 1 year. The correlation between the levels of pNfL at baseline, the age at multiple sclerosis onset, and a treatment response at M12 is consistent with a better effect in less disabled patients.

## Graphical abstract

MD1003 treatment was associated with clinical, brain and cervical spinal cord volumes improvement at 1 year. The correlation between the levels of pNfL at baseline, the age at MS onset and a treatment response at M12 is consistent with a better effect in less disabled patients. Our study provides a set of objective assessments that, taken together, argue for a possible neuroprotective or remyelinating effect of MD1003 in progressive forms of MS.

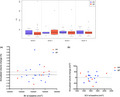

## 1 INTRODUCTION

Biotin is a water‐soluble vitamin widely present in small amounts in natural foodstuffs, in which it is mostly protein‐bound. It acts as a coenzyme of four important carboxylases, involved in gluconeogenesis, fatty acid synthesis, and the catabolism of several amino acids. MD1003 is an oral formulation of high‐dose pharmaceutical‐grade biotin (10,000 times the recommended daily intake) and is available in France under specific authorization. MD1003 leads to an increase in energy production in neurons and astrocytes, an increase in the production of citrate required for lipids synthesis, and the activation of acetyl CoA carboxylase 1 and 2, the rate‐limiting enzymes in the synthesis of long‐chain fatty acids required for myelin synthesis in oligodendrocytes (Sedel et al., 2016).

Due to its potential actions on neuroprotection and remyelination, MD1003 has been used in the progressive form of multiple sclerosis (PMS) in three clinical trials, with mixed results. The pivotal trial with MD1003 was a phase 2/3, double‐blinded, randomized study, using one dose of MD1003 versus placebo during one year (MS‐SPI study). In that trial, significantly more patients (12.6%, i.e., 1 out of 8; *p* = .005) in the MD1003 arm than in the placebo arm had achieved an improvement in Expanded Disability Status Scale (EDSS) or 25‐foot walk time (25FWT) at m (M) 9, confirmed at M12 (Tourbah et al., 2016). Despite these interesting results, differences in a reduction in MS‐related disability were noted between patients not taking fampridine (12/58; 20.3%) and those taking fampridine (1/45; 2.3%). Furthermore, a study on optic neuritis in MS did not find a significant improvement in visual acuity compared to placebo in patients with chronic visual loss (Tourbah et al., 2018), whereas a pilot study reported encouraging results in terms of patients’ visual function (Sedel et al., 2015).

Consequently, the effect and the mechanism of action of MD1003 remain elusive and further approaches are needed to try to resolve this point. In this study, we analyzed the clinical, biological, and MRI course of a cohort of patients with PMS treated with MD1003 and tried to identify biomarkers of treatment response.

## 2 MATERIALS AND METHODS

### 2.1 Patients

Patients were recruited from two referral hospitals in France, in Strasbourg and Dijon. A prospective cohort of 48 patients with PMS was recruited from January to June 2017 and followed during one year after MD1003 onset (100 mg twice a day). Inclusion criteria were men or women aged 30–70 years, evidence of disease progression with a significant increase of the EDSS score during the previous 2 years without any relapses during the last year, a primary or secondary PMS that fulfilled the revised 2017 McDonald criteria (Thompson et al., 2018) and a baseline EDSS score between 4 and 8.5. Patients with other neurodegenerative diseases and patients treated with immunosuppressive drugs introduced in the previous 3 months or with fampridine 1 month before inclusion or during follow‐up were not eligible for the study. All participants provided written informed consent at enrollment.

### 2.2 Plasma neurofilament light chain

Plasma samples were collected on the same day as the clinical visit and stored at −80°C following standard procedures (Teunissen et al., 2009). The plasma neurofilament light chain (pNfL) level in longitudinal serum samples was measured by SIMOA assay as previously described (Disanto et al., 2017). Inter‐assay coefficients of variation for three native serum samples were 4.5%, 6.7%, and 8.9% for control samples with mean concentrations of 7.3, 21.6, and 87.1 pg/ml, respectively. The mean intra‐assay coefficient of variation of duplicate determinations for concentration was 4.2%.

### 2.3 Cervical spinal cord volume, brain volume, and lesion load

Cervical spinal cord volume (CSCV) was assessed using an established semiautomatic cord image analyzer software package (CORDIAL) with minimal user–software interaction (Amann et al., 2016). CORDIAL combines a continuous max flow approach with spinal cord (SC) surface reconstruction that locates the boundary of the SC based on image voxel intensities in T1‐weighted MR images. Two cutting planes perpendicular to the SC centerline are determined based on predefined distances to an anatomical landmark, and the CSCV in between these boundaries is then calculated. The segmentation was performed over a 25‐mm‐long SC segment, starting 38 mm below the cisterna pontis. Segmentations were visually inspected for quality and were excluded from further statistical analysis in the case of segmentation errors.

The brain volume (BV) change between the baseline and M12 examinations was estimated with SIENA (Smith et al., 2001, 2002), using a robust brain center estimation. Cross‐sectional measures of the brain tissue volume (including separate estimates of volumes of gray matter, white matter, peripheral gray matter, and ventricular CSF), normalized for subject head size, were performed with SIENAX (Smith et al., 2001, 2002). Both steps were performed on the 3DT1w MRI.

Lesion load was evaluated on the FLAIR images by the Lesion Prediction Algorithm as implemented in the LST toolbox version 3.0.0 for SPM.

### 2.4 Experimental design

**FIGURE 1:**
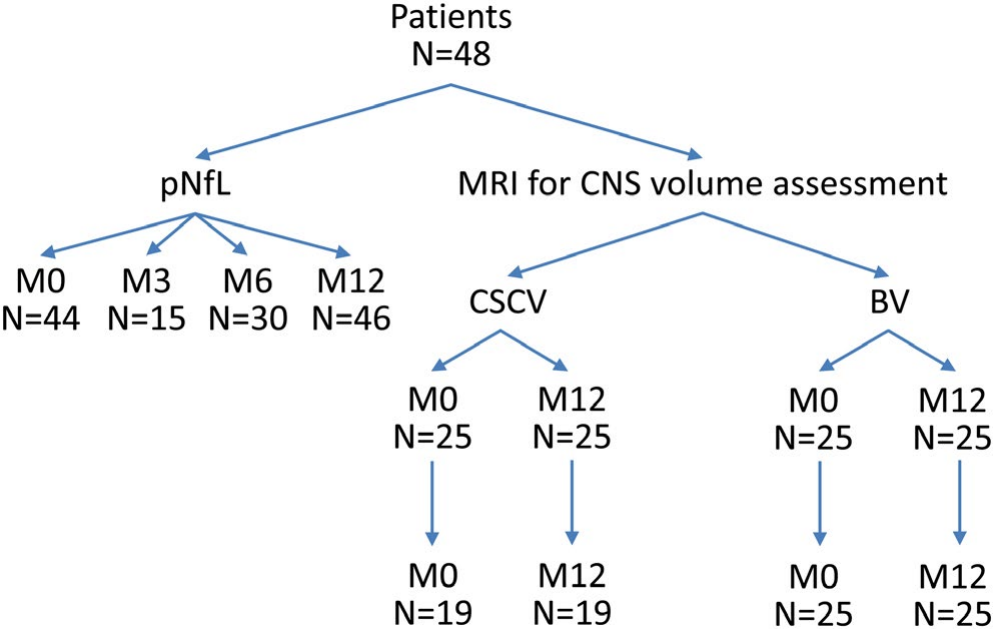
Flowchart of the biomarker assessments in the MD1003 cohort. BV, brain volume; CSCV, cervical spinal cord volume; M, month; pNfL, plasma neurofilament light chain; PP, primary progressive; SP, secondary progressive. pNFL level is expressed in pg/ml

The clinical assessment, including the EDSS score, the 25FWT, and the nine‐hole peg test (9HPT) score, was performed at M0, M3, M6, and M12. The pNfL level in blood at M0, M3, M6, and M12 was determined from blood samples. Spinal cord and brain volumes were quantified at M0 and M12. The number of patients analyzed for pNfL level and spinal cord/brain volume is shown in Figure 1. Treatment response at M12 was defined as a decrease of ≥0.5 points or ≥1 point in EDSS score (if baseline score was above 6 or 4.5–5.5, respectively), or a ≥20% decrease in 25FWT or 9HPT compared with the corresponding values recorded at baseline (inclusion visit). EDSS was assessed using Neurostatus EDSS (www.neurostatus.net) by EDSS raters qualified to Neurostatus level C. The mean of the two values of 25FWT and 9HPT for the dominant and nondominant hand achieved at each visit was recorded, except if the patient's disability status (EDSS > 7) precluded performance of these tests.

### 2.5 Statistical analysis

Categorical and ordinal variables were expressed as counts and percentages; continuous variables were expressed as mean, median and variances, range, and interquartile ranges (IQRs). Clinical score improvement was modeled with logistic regressions. Evolution in CSCV and BV throughout follow‐up and differences between secondary PMS (SPMS) and primary PMS (PPMS) patients were analyzed with linear models. pNfL levels, BV (including normalized gray and white matter volumes), CSCV, EDSS scores, 9HPT scores, and 25FWT, considering the temporal evolution through follow‐up, were modeled using linear mixed models with a time effect, a group or covariate effect, a time group or covariate interaction, and a random subject effect. Analyses were run on available data, and no missing data imputation was performed. All the statistical analyses were performed under the Bayesian paradigm. Results are expressed as 95% credibility interval (CI) on posterior distribution. Lowly prior information was used in every model. For logistic regression, the prior distribution for the log odds ratio (OR) was *N* (0, 2.34) that specified that the OR was a priori between 1/20 and 20. In linear models, the parameter prior distribution was *N* (0, 1,000). Note that this prior was applied after rescaling where necessary, that is, when the data range was larger than 1,000. Markov Chain Monte Carlo (MCMC) algorithms were used. MCMC chain convergence was assessed and verified graphically in all cases. Significant results were defined by a probability of odds ratio Pr(OR > 1) or Pr(OR < 1) of at least 90%. Receiver operating characteristic (ROC) curves were performed to define the threshold for pNfL level and age at MS onset as a determinant of treatment response. All computations were done using R software (version 3.5.1) and with JAGS software.

### 2.6 Ethical statement

This study was approved by the medical ethics committee of each center and conducted in accordance with internationally recognized ethical standards. Written consent to collect and use anonymized clinical data was obtained from each patient before the study.

## 3 RESULTS

The main demographic and clinical characteristics of the patients are summarized in Table 1. Mean EDSS was reported in all patients and was stable at 6.5 ± 0.9 from M0 to M12 (*p = *.64). Due to the high disability score in patients of this cohort, mean 25FWT was reported in 29 patients and 9HPT in 23 patients. Mean 25FWT changed from 22.3 ± 17.4 s at M0 to 20.5 ± 19.3 s at M12 (*p = *.54). Mean 9HPT score in the dominant hand changed from 31.2 ± 18.8 s at M0 to 32.8 ± 20.3 s at M12 (*p = *.76). Thirteen patients (27%) improved when a combined criterion using EDSS score, 25FWT, and 9HPT score was considered (Table 2).

**TABLE 1:** Characteristics of the patients at MD1003 onset

	MD1003
Female sex, *n* (%)	29 (60.4)
Age, mean (*SD*) (years)	59.9 (8.7)
Disease phenotype, *n* (%)	
PPMS	16 (33.3)
Age, mean (*SD*) (years)	63.8 (5.7)
SPMS	32 (66.6)
Age, mean (*SD*) (years)	58 (9.4)
Duration of MS, mean (*SD*) (years)	23.3 (10.1)
EDSS	
Number of patients assessed	48
Mean (*SD*)	6.52 (0.91)
Median (range)	6.5 (4–8)
EDSS 4.5–5.5, *n* (%)	6 (12.5)
EDSS 6–7, *n* (%)	30 (62.5)
EDSS 7.5–8.5, *n* (%)	12 (0.25)
25FWT	
Number of patients assessed	29
Mean, seconds (*SD*)	22.3 (17.4)
Median, seconds (range)	18.8 (7.4–82.5)
9HPT, dominant hand	
Number of patients assessed	23
Mean, seconds (*SD*)	31.2 (18.8)
Median, seconds (range)	26.6 (18.9–116.8)
Concomitant DMT, *n* (%)	10 (20.8)
Mycophenolate mofetil	7 (14.5)
Azathioprine	1 (0.02)
Interferon beta‐1a	1 (0.02)
Methotrexate	1(0.02)
Treatment with fampridine or amifampridine, *n* (%)	23 (47.9)

Abbreviations: 25FWT, 25‐foot walk time; 9HPT, nine‐hole peg test; DMT, disease‐modifying therapy; EDSS, Expanded Disability Status Scale; MS, multiple sclerosis; PPMS, primary progressive multiple sclerosis; *SD*, standard deviation; SPMS, secondary progressive multiple sclerosis.

**TABLE 2:** Characteristics of the patients with a treatment response under MD1003 defined by an improvement in one of the following criteria from month 0 (M0) to month 12 (M12): EDSS score, 25FWT, or 9HPT

Patients	Gender	Age at MS onset, year	MS form	Duration of the disease before MD1003 onset, year	DMT	Fampyra	EDSS at M0	EDSS at M12	Relapse (delay from MD1003 onset, m)	pNfL level at M0	pNfl level at M12	Mean Change in 25FWT (second, %)	Mean Change in 9HPT (second, %)	Mean annualized % change in BV	Mean annualized % change CSCV
1.	M	24	SP	28	–	0	8.5	8	0	20.4	27.3	NA	NA	NA	NA
2.	M	37	PP	40	–	1	7.5	7.5	0	49.6	47.3	NA	−9.8 (−23)	NA	NA
3.	M	18	SP	36	–	0	7.5	6.5	0	27.7	27.3	NA	NA	NA	NA
4.	M	24	SP	22	–	1	6.0	6.0	0	NA	29.5	−25.90 (−57)	−3.3 (−15)	−1.57	3.32
5.	F	21	SP	17	–	1	6.5	6.0	0	10.9	22.7	−10.77 (−21)	3.2 (+13)	NA	−0.73
6.	F	41	PP	26	–	1	7.0	6.0	0	56.7	35.4	−5.90 (−19)	−3.9 (−13)	−1.75	−1.93
7.	M	30	SP	29	MMF	1	8.5	8.0	1 (8)	31.5	34.1	NA	NA	3.14	NA
8.	F	42	SP	14	–	0	6.0	4.5	1 (8)	29.8	46.3	−0.45 (−6)	−0.1 (−0.2)	−0.43	2.91
9.	F	31	SP	27	–	0	6.0	6.0	0	27.7	32.0	−5.40 (−24)	−6.1 (+17)	−0.88	1.34
10.	F	48	PP	17	MTX	1	6.5	6.5	0	20.2	28.0	−10.73 (−38)	−4.3 (−17)	−0.07	NA
11.	F	35	SP	25	–	0	5.0	5.0	0	23.3	39.1	−3.66 (−32)	2.0 (+10)	−1.22	−0.09
12.	M	41	SP	18	–	1	6.0	4.0	0	16.1	20.8	−3.00 (−32)	−0.1 (−0.4)	1.03	NA
13.	M	39	SP	20	–	0	5.5	6.5	0	30.3	47.8	−2.47 (−24)	1.2 (+4.5)	−0.68	NA

Abbreviations: 25FWT, 25‐foot walk time; 9HPT, nine‐hole peg test; BV, brain volume; CSCV, cervical spinal cord volume; DMT, disease‐modifying therapy; EDSS, Expanded Disability Status Scale; MMF, mycophenolate mofetil; MS, multiple sclerosis; MTX, methotrexate; NA, not available; PP, primary progressive; *SD*, standard deviation; SP, secondary progressive.

During the follow‐up, seven relapses were reported retrospectively by six SPMS patients and one PPMS patient, after a mean duration of the disease of 18.9 ± 10 months and a median time of 6 months (range: 4–12 months) after MD1003 onset. Two of them were under immunosuppressants during the MD1003 treatment period. None of them were treated with methylprednisolone during the relapses and all seven patients experienced a complete recovery from the neurological symptoms.

**FIGURE 2:**
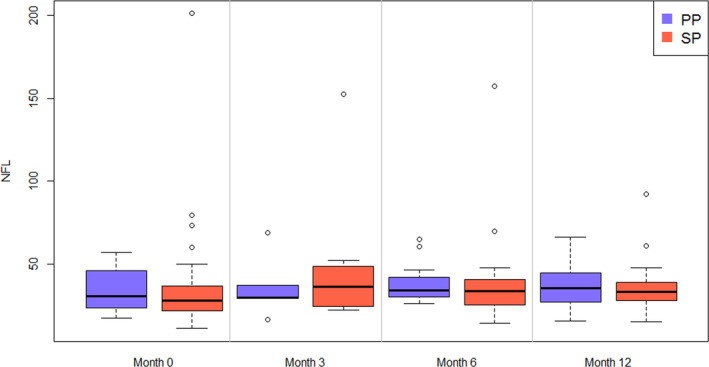
Course of the plasma neurofilament light chain level in progressive multiple sclerosis patients during the one‐year treatment with MD1003. PP, primary progressive; SP, secondary progressive. pNFL level is expressed in pg/ml

**FIGURE 3:**
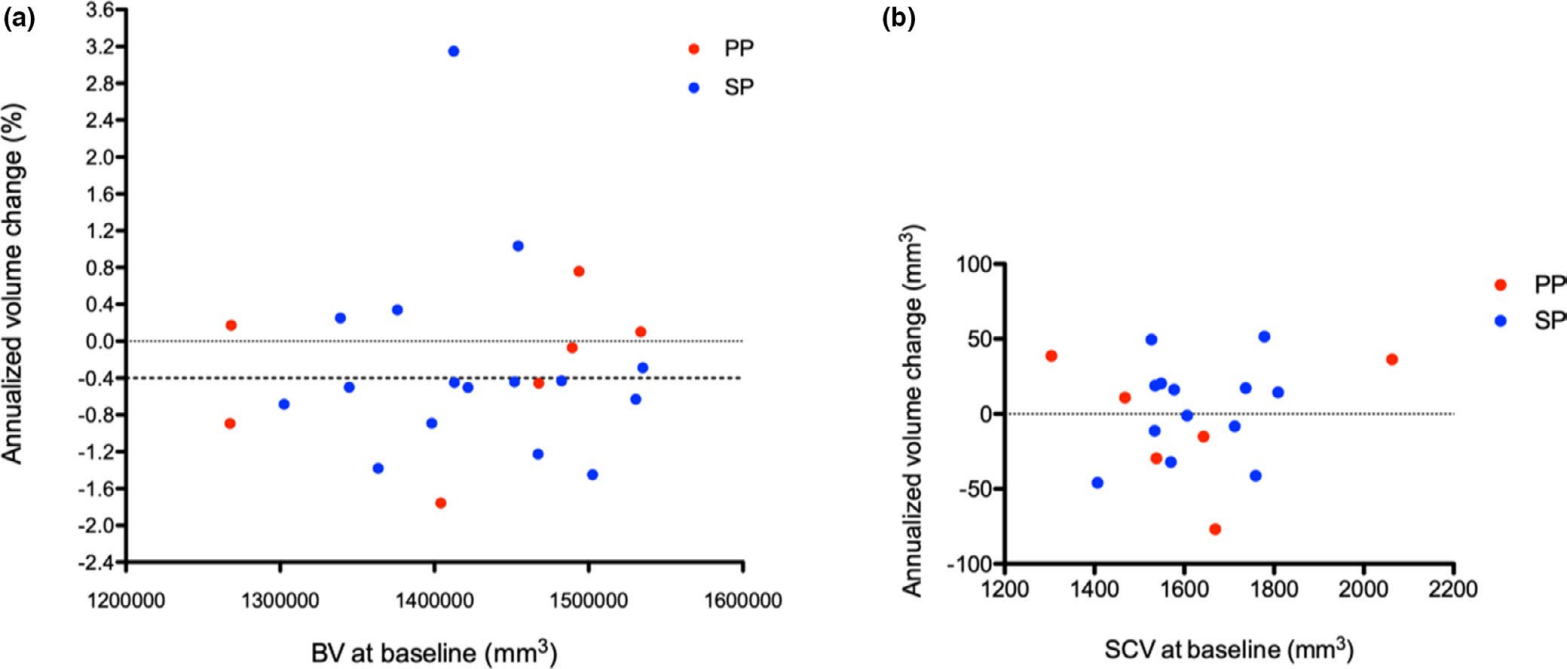
Mean annualized volume changes in brain (a) and in cervical spinal cord (b). Brain volume changes are expressed in % of change at M12 compared to baseline. Spinal cord volume changes are the comparison of volume in mm^3^ from baseline to M12. BV, brain volume; PP, primary progressive; SCV, spinal cord volume; SP, secondary progressive

The median pNfL level increased from baseline (28.5 pg/ml; range [10.9–79.4]) to M3 (35.3 pg/ml; range [16.2–152.3]), thereafter decreasing at M6 (33.6 pg/ml; range [13.9–157.2]) and at M12 (33.3 pg/ml; range [14.9–92.1]). At each time point, there was no difference between PMS subtypes (Figure 2). BV showed a mean annualized change of −0.27 ± 1% [range: −1.7% to 3.1%] between time points (Figure 3a). CSCV was available for 19 patients at baseline and M12, and showed annualized changes of between −5.1% and 3.5% with a mean of 0.0% and a standard deviation of 2.3% (Figure 3b). At an individual level, a gain of volume was observed in 7/24 patients for BV and 10/19 patients for CSCV at M12.

**FIGURE 4:**
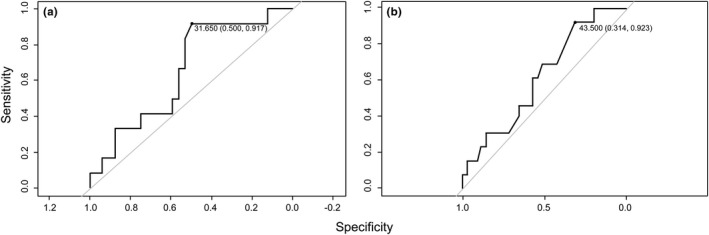
ROC curves determining the sensitivity and specificity for treatment response of the pNfL value at MD1003 onset and mean age at MS onset. The indicated point is the optimal cutoff point with the value of sensitivity and specificity associated. Value of pNfL lower than 31.65 pg/ml and age at MS onset lower than 43.5 years were both associated with a higher probability of response to MD1003. pNfL, plasma neurofilament light chain; ROC: receiver operating characteristic

Bayesian linear and logistic regressions were used to evaluate potential prognostic factors of treatment response at M12 (Table 3). A high pNfL level at MD1003 onset (OR 0.96; 95% CI [0.91; 1]), high age at MS onset (OR 0.95; 95% CI [0.89; 1.01]) and an increase in brain lesion load (OR 0.81; 95% CI [0.55; 1.05]) were the strongest predictors of the absence of a treatment response at M12. pNfL level and age at MS onset were both associated with a higher probability of response and the optimal thresholds were 31.65 pg/ml and 43.5 years, respectively, as determined by ROC curves (Figure 4). The role of the secondary progressive form in treatment response should be interpreted with caution according to the large credibility interval (OR 0.42; 95% CI [0.07; 1.27]). Normalized volume for gray and white matter at baseline and mean annualized changes in brain and cervical spinal cord volumes were not correlated with the treatment response at M12. High age was correlated with an increase of pNfL at M12 as well as a high EDSS score and an increase in CSCV at M12 (Table 4). We did not identify a specific role of clinical relapses or annualized change in brain lesion load as predictive factors for pNfL changes after 1 year of MD1003 treatment.

**TABLE 3:** Prognostic factors for improvement in EDSS score, 25FWT, or 9HPT score versus stabilization or worsening in patients treated with MD1003 during one year

Data assessed	OR	95% credibility interval	Probability for OR > 1
Gender (men)	2.36	0.58; 6.49	.861
Age at MS onset	0.95	0.89; 1.01	.077
Age at MD1003 onset	0.96	0.91; 1.03	.150
Duration of the disease before MD1003 onset	1.01	0.95; 1.08	.667
MS form (SP)	0.42	0.07; 1.27	.054
Relapse	1.36	0.18; 4.59	.508
Fampyra M0	1.57	0.39; 4.31	.666
IS M0	0.83	0.12; 2.67	.277
Clinical scores at baseline			
25FWT M0	1.00	0.95; 1.04	.554
9HPT score M0	0.95	0.86; 1.01	.106
EDSS score M0	1.13	0.62; 1.90	.621
MRI at baseline			
CSCV M0*	1.23	0.07; 5.93	.360
BVN M0**	1.05	0.68; 1.54	.551
GMV M0**	1.09	0.48; 2.15	.520
WMV M0*	1.17	0.49; 2.39	.582
BLL M0***	1.52	0.39; 4.13	.665
MRI annualized changes			
ACSCVC	1.40	0.83; 2.39	.882
ABVC	1.12	0.39; 2.53	.512
ABLLC	0.81	0.55; 1.05	.064
pNfL M0	0.96	0.91; 1.00	.053

Note

The statistical model used is a logistic regression.

Abbreviations: 25FWT, 25‐foot walk time; 9HPT, 9‐hole peg test; ABLLC, annualized brain lesion load change; ABVC, annualized brain volume change; ACSCVC, annualized cervical spinal cord volume change; BLL, brain lesion load at baseline (*** OR for 10,000 units increase); BVN, brain normalized volume at baseline (** OR for 100,000 units increase ); CSCV, cervical spinal cord volume at baseline (* OR for 1,000 units increase); EDSS, Expanded Disability Status Scale; GMV, gray matter volume at baseline (** OR for 100,000 units increase); IS, immunosuppression; M0, month 0; OR, odds ratio; pNfL, plasma neurofilament light chain; SP, secondary progressive; WMV, white matter volume at baseline (** OR for 100,000 units increase).

**TABLE 4:** Prognostic factors for plasma neurofilament light chain (pNfL) values in patients treated with MD1003 during one year

Data assessed	Coefficient	95% credibility interval	Probability for coefficient > 0
Gender (men)	2.59	−7.81; 13.13	.686
Age at MS onset	0.85	0.38; 1.33	1.000
Age at MD1003 onset	0.85	0.37; 1.32	1.000
Duration of the disease before MD1003 onset	0.70	0.23; 1.18	.998
MS form (SP)	−1.25	−12.21; 9.77	.406
Relapse	5.73	−8.62; 19.86	.789
Fampyra	0.593	−9.62; 10.77	.547
IS	2.01	−10.48; 14.71	.626
Clinical scores at baseline			
25FWT M0	0.04	−0.22; 0.32	.634
9HPT M0	0.03	−0.18; 0.25	.631
EDSS M0	6.36	1.67; 11.12	.996
Course of clinical scores			
25FWT	0.03	−0.26; 0.33	.602
9HPT score	0.06	−0.12; 0.24	.744
EDSS score	0.05	0.01; 0.10	.995
MRI at baseline			
CSCV M0*	−0.05	−18.21; 19.18	.492
BVN M0**	−0.30	−4.23; 3.68	.436
GMV M0**	−1.12	−8.34; 6.41	.379
WMV M0*	0.02	−7.18; 7.59	.497
BLL M0***	5.26	−2.93; 13.55	.899
MRI annualized changes			
ACSCVC	1.44	0.02; 2.83	.977
ABVC	1.37	−4.28; 7.02	.693
ABLLC	0.18	−1.32; 1.69	.595

Note

The statistical model used is a mixed linear model. Coefficient is the coefficient of the mixed linear model.

Abbreviations: 25FWT, 25‐foot walk time; 9HPT, 9‐hole peg test; ABLLC, annualized brain lesion load change; ABVC, annualized brain volume change; ACSCVC, annualized cervical spinal cord volume change; BLL, brain lesion load at baseline (*** OR for 10,000 units increase); BVN, brain normalized volume at baseline (** OR for 100,000 units increase ); CSCV, cervical spinal cord volume at baseline (* OR for 1,000 units increase); EDSS, Expanded Disability Status Scale; GMV, gray matter volume at baseline (** OR for 100,000 units increase); IS, immunosuppression; M0, month 0; OR, odds ratio; SP, secondary progressive; WMV, white matter volume at baseline (** OR for 100,000 units increase).

## 4 DISCUSSION

This study aimed to identify clinical, radiological, and biological biomarkers of treatment response to high‐dose pharmaceutical‐grade biotin (MD1003) in 48 patients with PMS. Twenty‐seven percent of patients fulfilled the criteria for treatment response at 12 months, based on a combined criterion using EDSS score, 25FWT, and 9HPT score. By applying Bayesian linear and logistic regression, we identified high pNfL level at MD1003 onset, high age at MS onset, and the increase in brain lesion load during MD1003 treatment as the strongest predictors of poor treatment response to MD1003. We observed that BV and CSCV were relatively stable over the one year of the study, a finding that may not correspond to the natural history of PMS and could therefore be an additional argument for a neuroprotective or remyelinating action of the drug (Baecher‐Allan et al., 2018). Finally, concurrent treatments with fampridine or immunosuppressive/immunomodulatory drugs did not affect the clinical treatment response.

Assessment of the treatment response was based on the endpoint used in the MS‐SPI phase 2/3 trial with MD1003 (Tourbah et al., 2016). Our proportion of responders was close to that reported in the MS‐SPI study, except that we did not confirm an improvement at 3 months. In this population of progressively disabled patients, the improvement of EDSS scores or 25FWT allows a sensitive assessment of the successive steps of disability progression (van Munster & Uitdehaag, 2017). In practical terms, from baseline EDSS score 0 to EDSS score 4, the impact of the treatment is mainly expressed by functional system scores. Beyond EDSS score 4, where ambulation is impaired, the 25FWT is more sensitive to capture some gait improvement. Furthermore, assessing walking speed should be clinically relevant, because it relates to the capacity to perform activities important in daily life (van Munster & Uitdehaag, 2017). As observed in the pivotal MS‐SPI study, 9HPT score improvement occurs very rarely in a 12‐month period of treatment with MD1003, but could be of high interest in patients who have lost the ability to walk. Our population was more disabled with a longer duration of the disease before MD1003 onset when compared to the patients included in the SPI trial and in other small series, which explains why we added this endpoint to our clinical assessment (Birnbaum & Stulc, 2017; Sedel et al., 2015; Tourbah et al., 2016).

We found that patients with MS onset before the age of 43 could have a better treatment response than those with MS onset after this threshold. Our results do not allow us to draw any conclusions on the role of the type of MS progression in the treatment response. The statistical trend for a poor prognosis in SPMS patients should be interpreted with caution because of a large credibility interval. Furthermore, this result should not be overinterpreted and the role of a primary progressive form in treatment response cannot be deduced from this. We identified a role of pNfL level in the treatment response. NfL is a marker of axonal loss and correlated significantly with inflammatory markers for MS activity, markers of neurodegeneration such as brain volume loss and therapeutic response (Kuhle et al., 2019). In our study, the course of the mean level of pNfL showed an increase at M3 and a slow decrease until M12, whereas the natural history of pNfL in the placebo groups of PMS shows stabilization or a regular increase over time (Kapoor et al., 2020). In addition, the course of the mean BV and CSCV was stable throughout the year. It should be noted that, at an individual level and despite a mean global stability of mean volume changes, a gain of volume was observed in 7/24 patients for BV and 10/19 for CSCV at M12, which is also uncommon in PMS. Another explanation could lie in the exacerbation of inflammation under MD1003. This point is a matter of debate, because the studies on this topic are contradictory, for example, one showing a relationship (Branger et al., 2020) and another showing none (Mathais et al., 2020). Whatever the case, the increase in brain volume or in the level of pNfL was not associated with the occurrence of relapses in our study. Moreover, and in accordance with previous studies, we confirmed that age and EDSS score correlated with the pNfL level, which gives added reliability to our results (Barro et al., 2018; Disanto et al., 2017).

We did not identify a specific role of clinical relapses or annualized change in brain lesion load as predictive factors for pNfL changes after 1 year of MD1003 treatment. Possible explanations could lie in the small number of relapses and their good prognosis given that they were followed by a complete spontaneous recovery without any increase of the EDSS score. Unfortunately, the course of the lesion load in the cervical spinal cord, which might have explained the role of the increase in its volume in the increase of pNfL, was not assessed. Lastly, taken together, our findings suggest that MD1003 could be less effective in patients with more advanced neuronal degeneration, corresponding to patients with a high age and a high level of pNfL. Despite the negative results of the SPI2 phase 3 study (NCT02936037), this set of results is of interest regarding the possibility of an innate improvement of the disability in 9% of untreated patients with primary PMS (Tremlett et al., 2012). Despite limitations related to a regression to the mean phenomenon and the absence of concurrent measures such as MRI or functional scores, this report is consistent with our results showing the same prognostic factors of improvement, such as a young age or the presence of a moderate disability.

The absence of correlation between brain and spinal cord volume changes and treatment response raises the challenging question of the assessment of the remyelinating process at the individual level. Quantitative diffusion imaging (DTI, NODDI), quantitative imaging of magnetization transfer (MTI, MTR, MPF), and myelin‐targeted PET radiotracers are recognized as being the most effective techniques for this purpose (Bodini et al., 2016; Chen et al., 2013; Zhang et al., 2012).

In contrast to other studies using MD1003, we did not find that not taking fampridine or having a baseline EDSS score 4.5–5.5 was associated with a better therapeutic response (Tourbah et al., 2016). A possible explanation for this could be the homogeneity of the EDSS scores in our population of PMS patients at baseline. On the other hand, the correlation between a good treatment response and a relatively low pNfL level at baseline supports the notion that MD1003 may be more effective in less disabled PMS patients and may consequently be consistent with the results of the MS‐SPI study. The SPI2 phase 3 study did not show did not show any efficacy of the drug on disability in PMS patients by a primary endpoint similar to that of the MS‐SPI study (Tourbah et al., 2016). Nevertheless, it would be premature to go further in the analysis because the results of the SPI2 trial have not yet been presented at any congress and, as yet, no publication is available.

Bayesian statistics were used for our study. This approach represents an efficient tool to improve the interpretation of clinical trials, especially in a small sample such as ours (Wijeysundera et al., 2009). In addition, Bayesian methods are among the most relevant means of counteracting the numerous limits of classical methods relying on *p*‐values (Hubbard & Lindsay, 2008).

## 5 CONCLUSION

The clinical effect of MD1003 in regard to the absence of significant mean changes in pNfL level, BV, and CSCV over time and the absence of immunosuppressive/immunomodulatory or fampridine effect in our study argues in favor of a nonsymptomatic action of the drug. The correlation between the level of pNfL at baseline, the age at MS onset, and a treatment response at M12 is consistent with a better effect in less disabled patients, as determined in the pivotal MS‐SPI study. Our study provides a set of objective assessments that, taken together, argue for a possible neuroprotective or remyelinating effect of MD1003 in progressive forms of MS.

## CONFLICT OF INTEREST

N. Collongues has received honoraria for consulting or presentations from Biogen Idec, Almirall, Novartis, Merck Serono, LFB, Teva Pharma, Sanofi‐Genzyme, MedDay, and Roche and is a member of the Editorial Board of *Journal de la Ligue Française contre la Sclérose en Plaques*. The other authors have no conflict of interest to declare in relation to this paper.

## AUTHOR CONTRIBUTIONS

N. Collongues and J. de Seze: Major role in the conceptualization of the study, the acquisition, and interpretation of data. Draft and revision of the manuscript for intellectual content. J. Kuhle, C. Tsagkas, J. Lamy, N. Meyer, C. Barro, K. Parmar, M. Amann, J. Wuerfel, L. Kappos, and T. Moreau: Acquisition and interpretation of data and revision of the manuscript for intellectual content.

### Peer Review

The peer review history for this article is available at https://publons.com/publon/10.1002/brb3.1998.

## Data Availability

The data that support the findings of this study are available on request from the corresponding author. The data are not publicly available due to privacy or ethical restrictions.
